# Primary Myofibroblasts Maintain Short-Term Viability following Submucosal Injection in Syngeneic, Immune-Competent Mice Utilizing Murine Colonoscopy

**DOI:** 10.1371/journal.pone.0127258

**Published:** 2015-05-27

**Authors:** Hassan A. Khalil, Nan Ye Lei, Wenxian Nie, Michael S. Lewis, Matthias G. Stelzner, Martín G. Martín, James C. Y. Dunn, James Yoo

**Affiliations:** 1 Department of Surgery, UCLA David Geffen School of Medicine, Los Angeles, California, United States of America; 2 Department of Pathology & Laboratory Medicine, VA Greater Los Angeles Health System, Los Angeles, California, United States of America; 3 Department of Surgery, VA Greater Los Angeles Health System, Los Angeles, California, United States of America; 4 Division of Gastroenterology and Nutrition, Department of Pediatrics, University of California Los Angeles, Los Angeles, California, United States of America; 5 Department of Surgery, Tufts Medical Center, Boston, Massachusetts, United States of America; Ghent University, BELGIUM

## Abstract

The myofibroblast is an important stromal cell of the gastrointestinal tract. Current *in vitro* and *in vivo* models either do not accurately recreate stromal-epithelial interactions or are not specific to myofibroblasts. We sought to create an animal model that would allow the study of myofibroblast-epithelial interactions. We isolated and cultured colonic myofibroblasts from FVB mice. Cells were α-SMA and vimentin positive but desmin negative on immunoblot analysis. We injected the myofibroblasts into the colonic submucosa of syngeneic adult mice (n = 8) via a miniendoscopic system. We then isolated green fluorescent protein (GFP) positive colonic myofibroblasts from C57BL/6-Tg(CAG-EGFP)1Osb/J mice and injected them into the colonic lamina propria of C57BL/6J mice at 1x10^5^ (n = 14), 1x10^6^ (n = 9), or 5x10^6^ cells/mL (n = 4). A subset of mice were injected with serum-free media and ink without cells (n = 3). Mice underwent repeat endoscopy and euthanasia one or 7 days after injection. Colons were isolated and either fixed in 10% formalin or the inked sites were individually excised and lysed for DNA. We assessed the injection sites via histology and immunohistochemical stains for α-SMA and GFP. We used qPCR to quantify GFP DNA transcripts at the lamina propria injection sites. Submucosal injection of myofibroblasts resulted in the formation of a subepithelial wheal on endoscopy, which persisted to day 7. Myofibroblasts injected either into the submucosa or lamina propria maintained viability on post-injection day 7 as evidenced by positive α-SMA staining. qPCR of lamina propria injections showed a dose-dependent increase in GFP DNA transcripts on post-injection day 1, whereas the number of transcripts on day 7 was equivalent for the concentrations injected. We demonstrate short-term survival of primary cultured colonic myofibroblasts in syngeneic mice. This may prove to be a valuable model for studying the role of myofibroblasts in states of health and disease.

## Introduction

The myofibroblast is an important stromal cell of the gastrointestinal (GI) tract that is believed to be involved in the regulation of many physiologic processes [1,2] ranging from intestinal stem cell differentiation and migration along the crypt-villus axis, mucosal repair, fibrosis, and the development of cancer [3–6]. The signaling mechanisms that regulate myofibroblast function have been studied *in vitro*, using both cell lines and primary cells taken from murine and human colon [1,2,7]. However, few *in vivo* models exist that allow the study of myofibroblast signal modulation on the overlying epithelium directly.

In-depth analysis of myofibroblast physiology requires the ability to not only study these cells *in vitro*, but to evaluate how changes in myofibroblast function impact surrounding cell populations and the overall function of the GI tract in an *in vivo* setting. Existing cell culture and animal models are limited in their ability to effectively study cell-cell interactions. *In vitro* co-culture models are often unable to recreate the interactions found in nature accurately, they poorly mimic actual physiologic conditions, and underestimate the contributions of the normal bowel wall architecture and surrounding cell populations that are essential elements of the GI microenvironment [5]. Animal models that utilize conditional gene targeting are neither organ- nor cell type-specific [8–10], since myofibroblasts lack a unique cell marker [8,9].

Unlike other GI tract organs, the mouse colon is accessible by endoscopy for evaluation of its mucosal surface without the need for surgical procedures or the sacrifice of animals. Endoscopy is not limited to visual inspection of the bowel wall, but provides a means for tissue sampling and other interventional procedures that are commonly performed in human patients. Utilizing a small animal colonoscope, the goal of our study was to develop a minimally invasive, reproducible, and well-tolerated technique for subepithelial implantation of primary myofibroblasts into the colon wall of live, immune-competent, syngeneic mice.

In this study we describe a novel technique that has the potential to allow for the real-time *in vivo* study of stromal-epithelial cell interactions. The technique first involves the isolation of primary myofibroblasts from mouse colon tissue, using a well-established technique [11]. These cells can be grown in cell culture, and they have been previously shown to maintain viability following subcutaneous injection into immune-compromised animals [5]. Based on these observations, we hypothesized that that primary myofibroblasts might survive following injection into the colon wall if we employed a technique that has been previously described using cancer cell lines [12]. In the present study, we utilized standard colonoscopic techniques to implant primary myofibroblasts into the submucosa and lamina propria of the mouse colon wall. We demonstrate that myofibroblasts can be successfully and reproducibly implanted in the colon wall and maintain short-term viability in immune-competent, syngeneic mice.

## Methods

### Myofibroblast Isolation and Culture

Mouse colonic myofibroblasts were isolated from male or female 8–12 week old FVB mice (Jackson Laboratory, Bar Harbor, ME) as previously described [13]. Briefly, the colon was washed with ice cold sterile PBS and then shaken five times for 15 min in HBSS containing 5 mM EDTA, which de-epithelialized the tissue. Next, the tissue was incubated in 20 ml of RPMI-5 [RPMI with 5% FCS, 10 mM HEPES, 2 mM L-glutamine, 1 mM sodium pyruvate, 100 U/ml Pen-Strep] containing 10.5 mg of dispase (GIBCO-Invitrogen, Carlsbad, CA) and 7.2 mg of collagenase D (Roche Diagnostics, Indianapolis, IN) for 2 h in a shaking 37°C incubator. The digested tissue was treated with ACK lysis buffer for 5 min, and then was passed through a 70-μm cell strainer into 100-mm dishes in RPMI-5. After a 3-hour incubation, the non-adherent cells were washed away; leaving adherent cells that consisted mainly of macrophages, epithelial cells, and myofibroblasts. After several days, the macrophages and epithelial cells died off leaving cells that were morphologically homogeneous and composed primarily of cells with a myofibroblast phenotype. These cells stained positive for both α-SMA and vimentin but negative for desmin on immunoblot analysis. For the *in vivo* experiments, primary myofibroblasts were used 1–2 weeks after initial isolation.

Myofibroblasts were also isolated from C57BL/6-Tg(CAG-EGFP)1Osb/J mice that constitutively express the green fluorescent protein (GFP), originally obtained from the Jackson Laboratory (Bar Harbor, ME) and bred at our institution.

### Development of an Endoscopic Approach for Subepithelial Myofibroblast Injections

**Fig 1:**
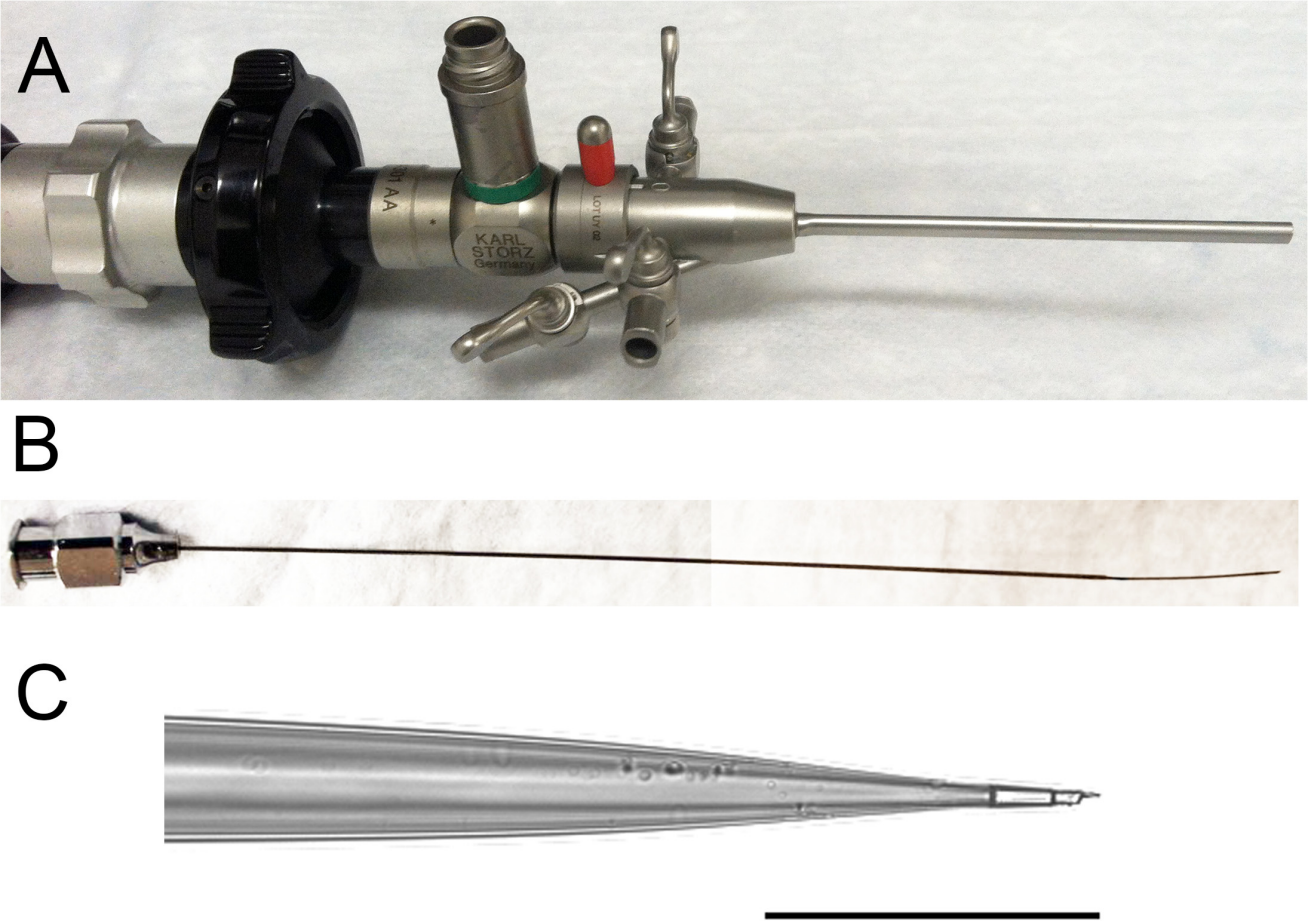
(A) Miniature endoscope. (B) Custom-made injection needle with a 30 gauge stainless steel tip. (C) Pulled and beveled capillary tubing for subepithelial injection. Scale bar 50 μm.

Endoscopic procedures were performed on anesthetized 8 to 12 week old male and female FVB (n = 8) or C57BL/6J (n = 30) mice using a 1.9 mm Karl Storz Coloview miniendoscopic system (Karl Storz, Tuttlingen, Germany, **Fig 1A**) after placing the mice on a water-only diet overnight. Anesthesia was induced with a vaporizer using 3% isoflurane which was decreased to 1% for maintenance.

For submucosal injections, a specially designed needle was created (**Fig 1B**) by brazing a 2-cm long 30-gauge needle to the end of a 10-cm long 26-gauge stainless steel needle (McMaster Carr, Santa Fe Springs, CA). The 30-gauge tip was small enough to reliably enter the submucosal space, while the 26-gauge extension provided enough length to pass through the working port of the endoscopic sheath.

For lamina propria injections, a micropipette was fabricated using glass capillary tubing with OD 0.84 mm and ID 0.60 mm (King Precision Glass, Claremont, CA), which was pulled with a vertical micropipette puller (David Kopf Instruments, Tujunga, CA) with a heated coil in a two-step fashion at 14 and 16 amperes, respectively, and fitted onto the metal needle. The tip diameter was approximately 2 μm wide (**Fig 1C**). The tip was beveled under a dissecting microscope using a computer hard drive spinning spun at 5,400 rpm that was radially etched with 600-grit sandpaper [14].

To confirm that injections could successfully be placed in the submucosa and lamina propria layers of the colon wall, initial experiments were performed using Spot (GI Supply, El Paso, TX), a sterile, biocompatible non-India-ink-based permanent carbon ink suspension used to “tattoo” the colon wall. Submucosal injections were carried out by inserting the tip of the 30-gauge needle into the colon wall. A submucosal wheal confirmed successful injection into the submucosal space. On the other hand, for successful lamina propria injections, the micropipette bevel was pushed against the colon wall while injecting and the presence of a punctate spot of ink was confirmed; presence of a wheal would suggest a deeper injection into the submucosa.

For subepithelial injection of myofibroblasts, cells were suspended in low glucose DMEM (Life Technologies) without serum. To facilitate later identification of the injection sites, 1.5% Spot was added to the cell suspensions. For submucosal injections, 100 μL of either DMEM (n = 4) or myofibroblast suspension at a concentration of 5x10^5^ cells/mL (n = 4) was injected. For lamina propria injections, the ventral rectum and colon were injected with several μL of either DMEM alone (n = 3) or GFP-positive myofibroblasts at a concentration of 1x10^5^ (n = 14), 1x10^6^ (n = 9), or 5x10^6^ cells/mL (n = 4).

After the procedure, all mice were monitored for appropriate recovery from anesthesia and placed back in standard laboratory mouse cages with full access to water and chow. Mice underwent repeat colonoscopy 1 or 7 days later and then were euthanized with isoflurane overdose and colon specimens were isolated.

### Whole-mount Fluorescence Microscopy

Colons of mice that had been injected with GFP-positive myofibroblasts were harvested, opened on the mesenteric aspect and placed onto an ice-cold glass slide with the mucosa facing downward. Ink-laden areas were examined using a fluorescent microscope.

### Histology and Immunohistochemistry (IHC)

Near-confluent myofibroblasts cultured on 0.95 cm^2^ wells were fixed with 10% buffered formalin for 10 minutes at room temperature prior to immunostaining. Isolated mouse colons were flushed with cold PBS then filled with 60 °C Histogel (American MasterTech, Lodi, CA) and fixed overnight in 10% buffered formalin solution. Specimens were embedded in paraffin and sectioned at 3 μm thickness. Hematoxylin and eosin (H&E) staining and IHC were performed per standard protocol. Heat-mediated antigen retrieval was performed in boiling Citra buffer (Fisher Scientific, Pittsburgh, PA) for 20 minutes. For biotinylated stains, sections were stained with prediluted monoclonal mouse anti-α-smooth muscle actin antibody (α-SMA), biotinylated secondary antibody, and streptavidin-horseradish peroxidase (all from Dako, Carpinteria, CA). For immunofluorescence, slides were stained with polyclonal rabbit anti-α-SMA (Abcam, Cambridge, MA) at 1:100 dilution, monoclonal rabbit anti-vimentin antibody (Abcam) at 1:250 dilution, monoclonal mouse anti-human desmin antibody (Dako) at 1:100 dilution, and chicken anti-green fluorescent protein antibody (Aves Labs, Tigard, OR) at 1:500 dilution. Primary antibodies were conjugated with 1:200 dilution of goat anti-rabbit or anti-mouse antibodies (Life Technologies, Grand Island, NY) or goat anti-chicken IgY antibody (Aves Labs). All antibodies were diluted in blocking solution consisting of 2% BSA and 4% normal goat serum. Slides were counterstained with 4',6-diamidino-2-phenylindole (DAPI; Life Technologies).

### Quantitative PCR

Injected areas of the colon identified by black ink staining were isolated and individually placed in 200 μL buffer ATL with 10% proteinase K (Qiagen, Valencia, CA) at 60 °C for at least 1 hour. DNA was extracted with the DNeasy blood and tissue kit (Qiagen). Quantitative PCR (qPCR) was performed using a GFP TaqMan probe (Life Technologies). To convert the qPCR data to number of GFP-positive DNA transcripts, a DNA ladder was constructed by performing qPCR on extracted DNA from a known number of GFP-positive myofibroblasts (range 1x10^2^ to 1x10^6^).

### Western Blot

Confluent mouse myofibroblasts were lysed in 2x SDS-PAGE sample buffer (20 mM Tris·HCl pH 6.8, 6% SDS, 2 mM EDTA, 4% 2-mercaptoethanol, and 10% glycerol) and boiled for 10 min. After SDS-PAGE, proteins were transferred to Immobilon-P membranes. The transfer was carried out at 100 V, 0.4 A at 4°C for 5 hours using a Bio-Rad transfer apparatus. The transfer buffer consisted of 200 mM glycine, 25 mM Tris, 0.01% SDS, and 20% CH_3_OH. For detection of proteins, membranes were blocked using 5% nonfat dried milk in PBS (pH 7.2) and then incubated for 2 hours with the desired antibodies diluted in PBS (pH 7.2) containing 3% nonfat dried milk. Primary antibodies bound to immunoreactive bands were visualized by ECL detection with horseradish peroxidase-conjugated anti-mouse or anti-rabbit antibodies (GE Healthcare, Piscataway, NJ).

### Data Analysis

Immunofluorescence and bright-field images were analyzed using ImageJ (National Institutes of Health, Bethesda, MD). PCR data was analyzed in Excel (Microsoft Corporation, Redmond, WA) and reported as mean ± standard deviation. Statistical analysis was performed with the Wilcoxon signed-rank with *p*<0.05 assumed to be significant.

### Ethics Statement

All animal studies were approved by the animal research committee at UCLA (ARC #2004–016, 2012–038, and 2012–077). The UCLA animal facility is accredited by the AALAC. This study was carried out in accordance with the recommendations in the Guide for the Care and Use of Laboratory Animals of the National Institutes of Health.

## Results

### Characterization of Colonic myofibroblasts

**Fig 2:**
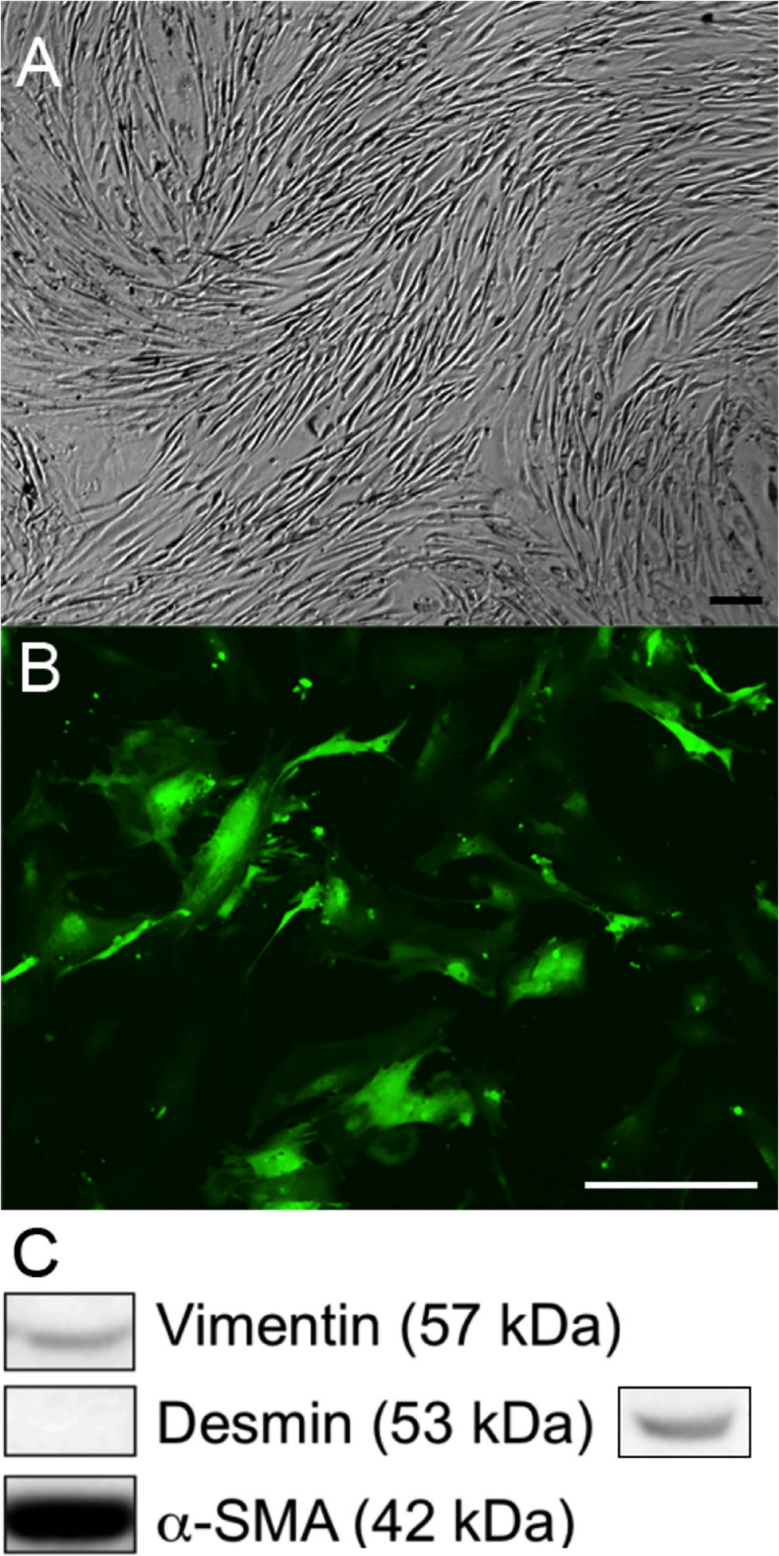
Cultured FVB mouse colonic myofibroblasts: (A) brightfield, (B) fluorescence microscopic image showing epifluorescence of GFP-positive myofibroblasts. (C) Western blot demonstrating positive vimentin and α-SMA stain and negative desmin stain of primary mouse colonic myofibroblasts. Positive control stain for desmin is shown to the right. Scale bar in A and B is 100 μm.

Primary mouse colonic myofibroblasts were isolated using a well-established technique [7,13,15]. Cultured colonic myofibroblasts took a stellate appearance when grown in culture (**Fig 2A and 2B**). Western blot analysis showed that cultured FVB mouse colonic myofibroblasts were positive for both α-SMA and vimentin while contamination with desmin-positive smooth muscle cells was low (**Fig 2C**). Colonic myofibroblasts cultured from C57BL6 mice showed the same expected staining pattern (**S1 Fig**). In this figure, the positive control stains were performed on small intestine.

### Endoscopic Model and Injection into the Submucosa and Lamina Propria

Mice tolerated the colonoscopy procedure well. The rate of colonic perforation associated with endoscopy was 3/41 (7.3%) and all cases occurred during the early phase of the study. These mice were euthanized immediately after the procedure and excluded from analysis. Successful endoscopic injections into the colon wall were performed using the custom-made systems described above (**Fig 1A-1C**).

**Fig 3:**
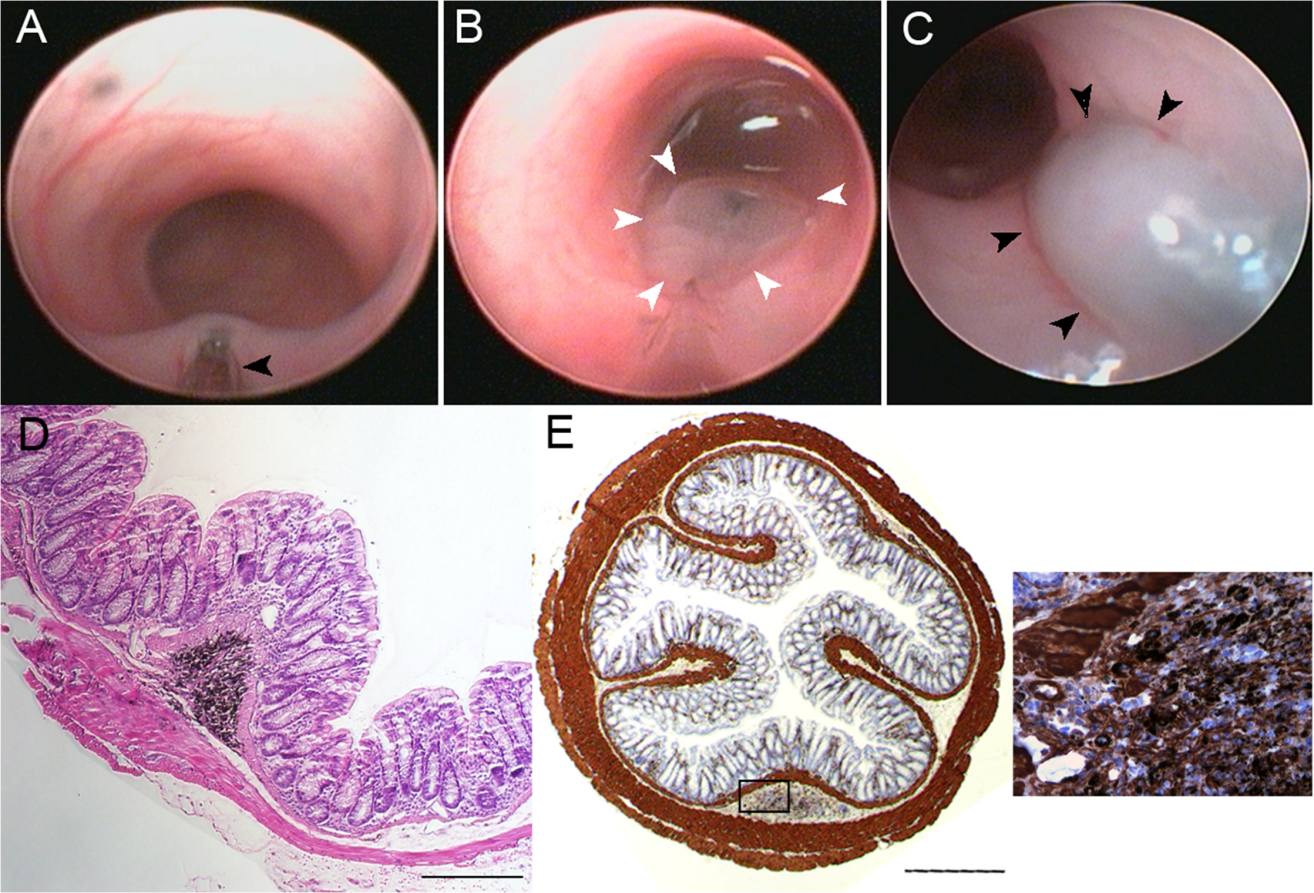
(A,B) Endoscopic view of mouse colon with a submucosally placed needle(black arrowhead; A) and immediately after injection (white arrowheads, B). (C) Endoscopic view of mouse colon 7 days post injection (black arrowheads). (D) H&E stain of mouse colon with 1x10^5^ cells/mL with ink injected endoscopically into the submucosa; scale bar 100 μm. (E) Anti-α-SMA IHC of submucosally injected myofibroblasts with high power insert; scale bar 500 μm.

**Fig 4:**
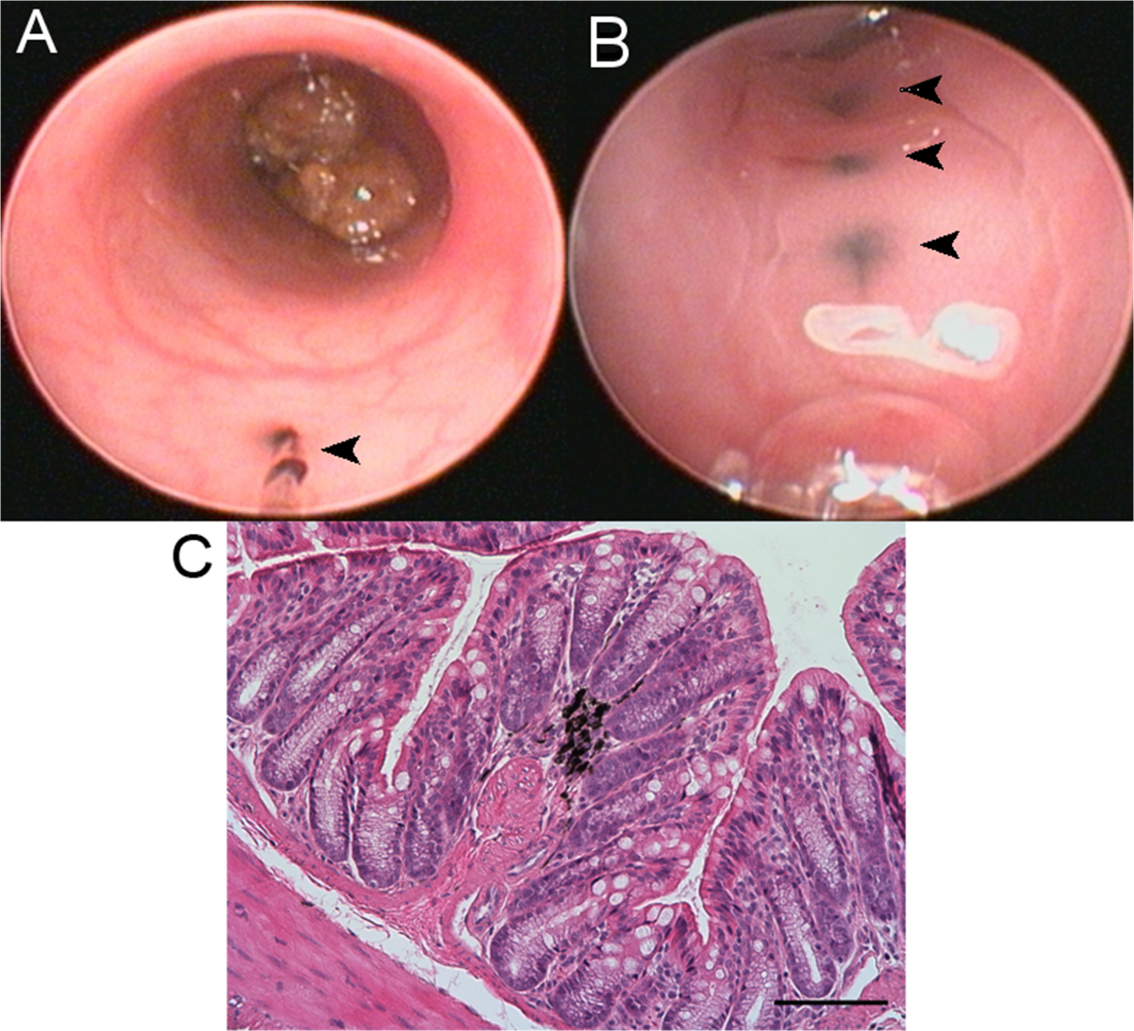
(A) Endoscopic view of mouse colon with ink injected into the lamin propria (black arrowhead). (B) Follow up colonoscopy 7 days later confirming presence of ink in the lamina propria (black arrowheads). (C) H&E stain of mouse colon showing presence of ink in the lamina propria; scale bar 100 μm.

Proper injection of Spot into the submucosal layer was visualized endoscopically by the appearance of a mucosal wheal (**Fig 3A and 3B**, **S1 Video**). Mice were re-scoped one week later, confirming that the site of injection could be easily identified (**Fig 3C)**. Injection into the submucosal layer was confirmed by subsequent histologic evaluation (**Fig 3D**). While the custom-made 30-gauge needle was able to reliably enter the submucosal space, the needle tip was too large for lamina propria injections. Therefore, capillary tubes were pulled and beveled, as outlined in Methods, to create a needle tip that was roughly 5 μm in diameter. Using this, lamina propria injections were also performed with Spot and were identified by the endoscopic appearance of sub-epithelial ink that did not wash away (**Fig 4A)**. As with the submucosal injections, the inked area could be readily re-identified upon repeat colonoscopy at 1 week (**Fig 4B**), and injection into the lamina propria was confirmed histologically (**Fig 4C**).

### Implantation of Primary Mouse Colonic Myofibroblasts

Primary mouse myofibroblasts were isolated and grown in cell culture, as described in Methods. After the addition of 1.5% Spot, myofibroblast cell suspensions were injected at 5x10^5^ cells/mL into the submucosa. Submucosal delivery of the injectate was confirmed with the observation of a submucosal wheal in real time. A follow up colonoscopy at 1 week identified an inked area with a submucosal bulge (**Fig 3C**). H&E staining of histology sections revealed that the normal bowel wall architecture was not disrupted at the injection site, there was minimal infiltration of immune cell populations (**Fig 3D**), and the cells within this inked submucosal region stained positively for α-SMA (**Fig 3E**), suggesting successful engraftment of the injected myofibroblasts. Overall, 3/4 (75%) of the injected specimens that were histologically analyzed showed positive engraftment in the submucosal position.

Lamina propria injections were also performed. Because of the small caliber of the injection system, 1x10^6^ cells/mL was the highest concentration that could be successfully injected. Higher cell concentrations plugged the tip of the needle. Also, a much smaller volume of cell suspension (approximately 1 μL) could be injected into the lamina propria. Mice received 2–3 injections at multiple axial levels. The inked areas could be re-identified on follow up endoscopy at 1 week but no mucosal bulge was visible (**Fig 4B**).

### Primary Mouse Myofibroblasts Maintain Short-Term Viability Following Injection into the Lamina Propria

**Fig 5:**
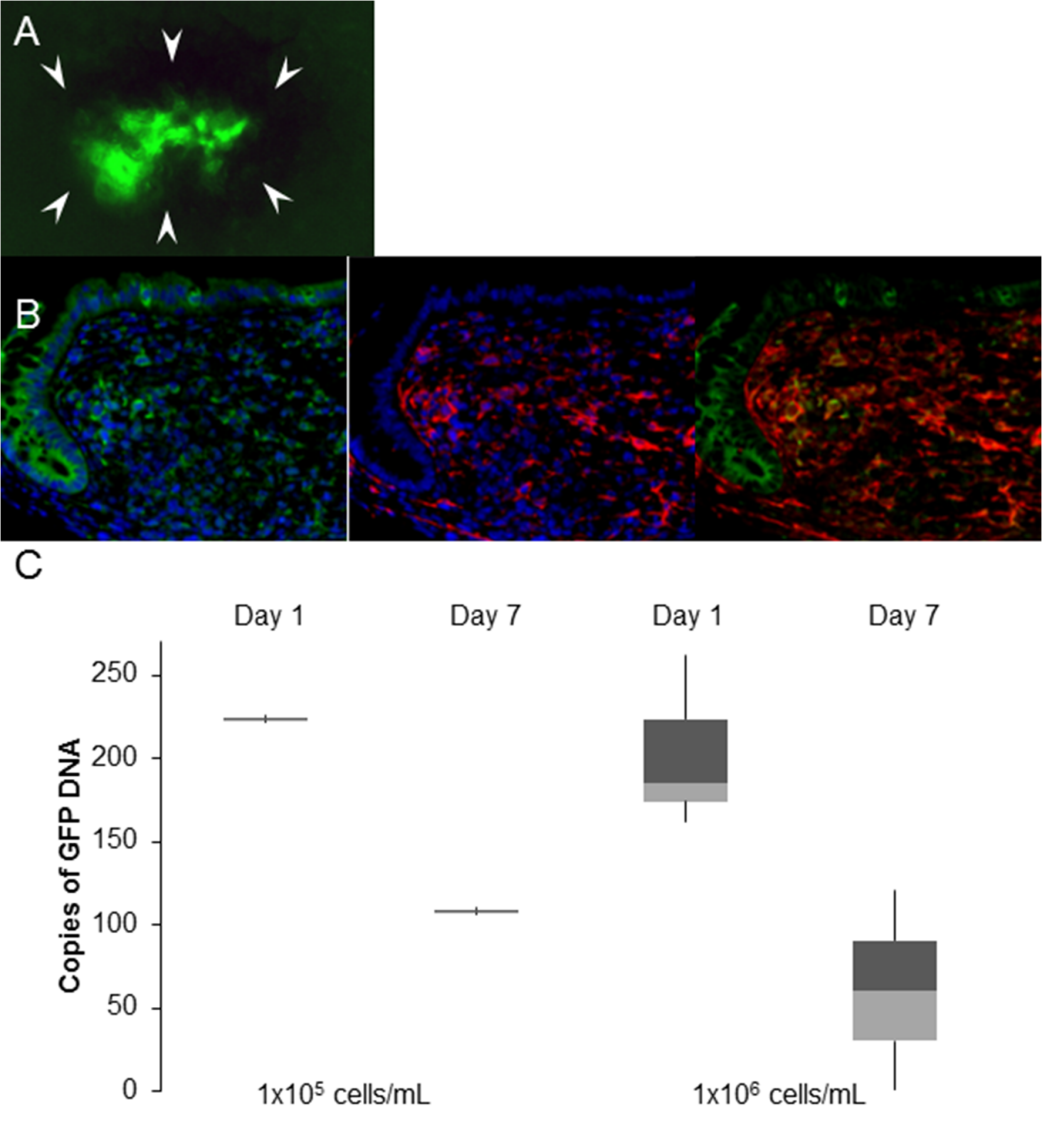
(A) Whole-mount epifluorescence of a segment of mouse colon injected with GFP-positive myofibroblasts surrounded by a rim of black ink (white arrowheads). (B) Anti-GFP (green) and anti-α-SMA (red) stains of mouse colon injected with GFP-positive myofibroblasts; nuclei are counterstained with DAPI (blue); merged image shows GFP and α-SMA double-positive cells. Scale bar 100 μm. (C) qPCR quantification of GFP-positive myofibroblasts injected submucosally into mouse colon at two concentrations on post-injection days 1 and 7.

Having established that injections could be consistently performed in the submucosal layer of the mouse colon wall, and that α-SMA-positive cells were present one week following injection, we sought to implant myofibroblasts into the lamina propria just under the epithelium, where they normally reside. To definitively establish that the α-SMA-positive cells identified on histologic examination were the myofibroblast cells that we injected. To do this, GFP-positive myofibroblasts harvested from GFP-positive mice and were grown in cell culture (**Fig 2B)**. These cells were then endoscopically injected into wild-type C57BL6 mice, as described. Whole-mount fluorescent microscopy on post-injection day 7 revealed that the injection sites were indeed GFP-positive (**Fig 5A**). IHC staining of these sites confirmed the presence of cells that were double-positive for GFP and α-SMA (**Fig 5B**) in 5/12 (41.7%) histologic samples, excluding the technically unattainable 5x10^6^ cells/mL injections. We then quantified GFP DNA by qPCR and as expected, one day post-injection, there were more cells in the injection sites that receive a higher concentration of cells, but day 7 cell engraftment was similar among the concentrations tested (**Fig 5C; S1 Table)**.

## Discussion

Very few *in vivo* models exist that are capable of studying cell-cell interactions in their native tissue environment. Primary myofibroblasts can be harvested from mouse colon and grown in tissue culture [7,13,15], but re-implantation of primary myofibroblast cells into a syngeneic, immune-competent mouse has never been reported. We describe the successful endoscopic implantation of primary myofibroblasts that maintain short-term viability in the submucosal layer of the mouse colon wall.

The injection and growth of primary, non-cancerous cells was previously demonstrated by subcutaneous injection in immune-deficient mice [5]. We demonstrate that primary myofibroblasts can also survive when re-implanted in the wall of the mouse colon. The presence of native growth factors and an intact blood supply appear sufficient to support short-term growth. Interestingly, at 1 week post-injection the number of viable cells remained relatively constant regardless of the concentration of cells that were initially injected (**Fig 5C)**. Since these are primary cells and not cancer cell lines, these findings may suggest that the growth kinetics of injected primary cells plateau over time, regardless of the concentration of injected cells. However, our study is not statistically powered to address this subject and further studies are needed to evaluate the long-term viability and growth kinetics of the submucosally injected cells.

Endoscopic cell microinjection creates small patches of colon containing myofibroblasts that can be genetically altered prior to re-implantation. This was well-demonstrated with the GFP-positive myofibroblasts that were isolated from a transgenic mouse. This approach can be used to study how specific changes in myofibroblast function (i.e., cell signaling events, protein expression) affect the overlying epithelium under a variety of conditions. The technique allows for targeted manipulation of a subpopulation of myofibroblasts in a segment of mouse colon that can be easily re-identified, serially evaluated (both endoscopically and histologically), and directly compared to adjacent tissue under the same experimental conditions. The ability to inject multiple sites in the same animal allows for a direct comparison of a specific intervention in an identical host, as each animal will serve as its own control. This technique can be applied to any existing mouse model, and any mouse strain, to study the contribution of the myofibroblast to a variety of processes including the regulation of the intestinal stem cell niche, epithelial repair following injury, inflammatory bowel disease, and the development and progression of cancer [10,11].

While this approach shows promise, there were a number of limitations to the study. First, we demonstrate only short-term survival of colonic myofibroblasts; long-term survival including the possibility of differentiation and reverse differentiation of the primary myofibroblasts need to be carefully evaluated [7]. Use of GFP positive myofibroblasts will facilitate the identification of implanted cells in this setting, as demonstrated here. Future directions include functional assays to determine whether physiologic interactions occur between the implanted myofibroblasts and the overlying epithelium.

In conclusion, primary myofibroblasts are capable of re-implantation in the submucosal layer of the mouse colon wall and appear to remain viable in the short term. While long-term viability and functional studies are required, this approach shows promise as a tool to study stromal-epithelial interactions in an *in vivo* setting.

## Supporting Information

S1 Fig (A-C) Immunostaining of cultured C57BL/6-Tg(CAG-EGFP)1Osb/J mouse colonic myofibroblasts: (A) α-SMA, (B) vimentin, and (C) desmin, scale bar 200 μm. (D) Mouse small intestine stained with α-SMA and vimentin (scale bar 50 μm). (E) Mouse small intestine stained with α-SMA and desmin (scale bar 20 μm). Nuclei are counterstained with DAPI.
(TIF)

S1 Video Endoscopic injection in the submucosal layer of mouse colon. (MP4)

S1 Table GFP-positive ISEMFs injected at two concentrations into lamina propria quantified by qPCR on post-injection day 1 or 7. (DOCX)
