# Supplementary figures and images for: Primary Myofibroblasts Maintain Short-Term Viability following Submucosal Injection in Syngeneic, Immune-Competent Mice Utilizing Murine Colonoscopy
Source: PLoS One. 2015 May 27;10(5):e0127258. doi: 10.1371/journal.pone.0127258 (PMC4445916; doi:10.1371/journal.pone.0127258)

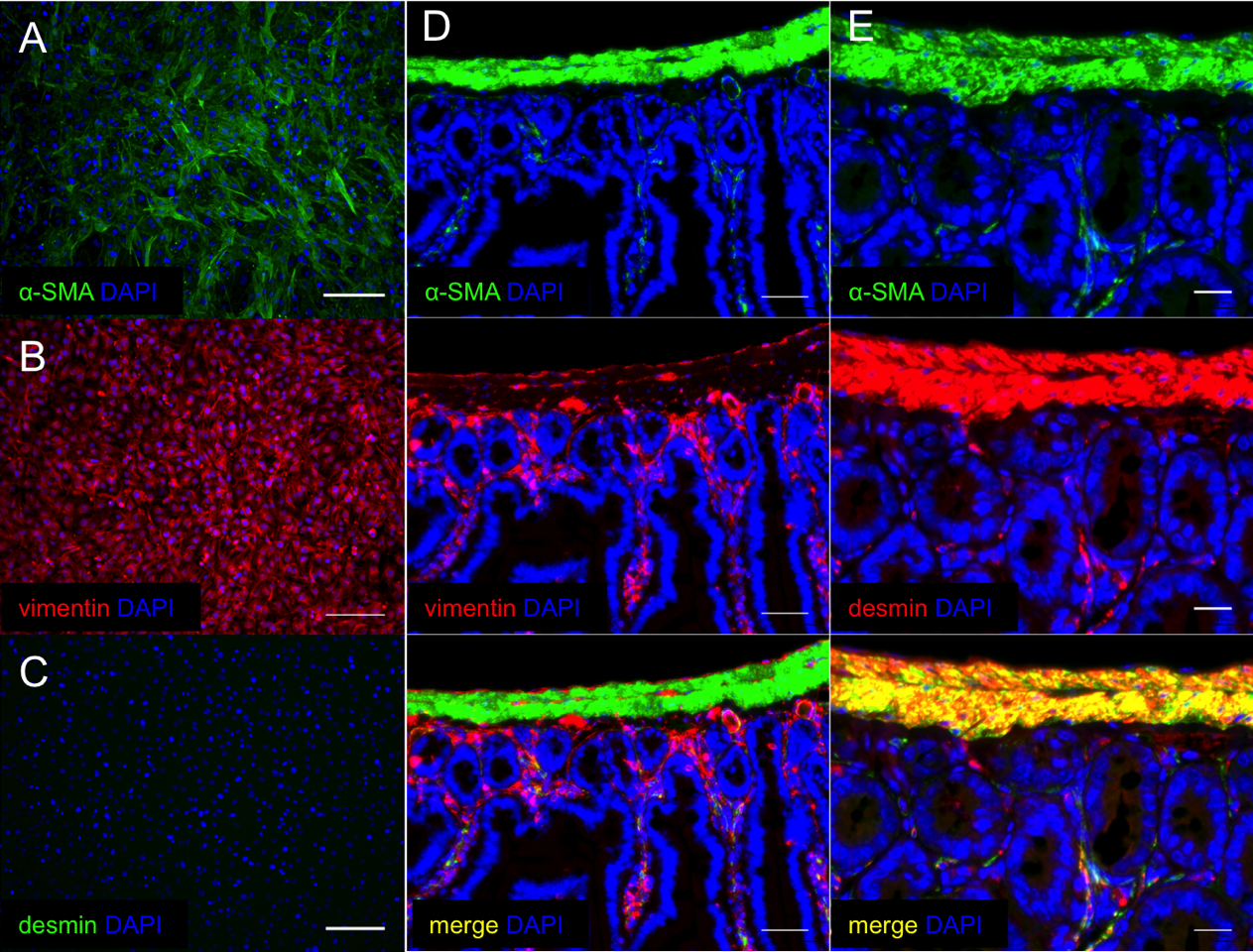

Supplement: S1 Fig — (D) Mouse small intestine stained with α-SMA and vimentin (scale bar 50 μm). (E) Mouse small intestine stained with α-SMA and desmin (scale bar 20 μm). Nuclei are counterstained with DAPI. (TIF) [file pone.0127258.s001.tif]
