# Supplementary material for: Primary Myofibroblasts Maintain Short-Term Viability following Submucosal Injection in Syngeneic, Immune-Competent Mice Utilizing Murine Colonoscopy
Source: PLoS One. 2015 May 27;10(5):e0127258. doi: 10.1371/journal.pone.0127258 (PMC4445916; doi:10.1371/journal.pone.0127258)
Supplement: S1 Table — (DOCX) [file pone.0127258.s003.docx]

**Supplementary Table.**

|  | **Concentration (cells/mL)** | |  |
| --- | --- | --- | --- |
|  | **1x10^5^** | **1x10^6^** | ***p*** |
| **Day 1** | 223.5±3.1 | 203.2±52.1 | >0.2 |
| **Day 7** | 108.08±2.9 | 60.4±85.4 | >0.2 |
